# Standardization of Size, Shape and Internal Structure of Spinal Cord Images: Comparison of Three Transformation Methods

**DOI:** 10.1371/journal.pone.0076415

**Published:** 2013-11-05

**Authors:** Yasuhisa Fujiki, Shigefumi Yokota, Yasumasa Okada, Yoshitaka Oku, Yoshiyasu Tamura, Makio Ishiguro, Fumikazu Miwakeichi

**Affiliations:** 1 Department of Statistical Science, School of Multidisciplinary Sciences, The Graduate University for Advanced Studies, Tachikawa, Tokyo, Japan; 2 Department of Anatomy and Morphological Neuroscience, Shimane University School of Medicine, Izumo, Shimane, Japan; 3 Department of Internal Medicine, Murayama Medical Center, Musashimurayama, Tokyo, Japan; 4 Department of Physiology, Hyogo College of Medicine, Nishinomiya, Hyogo, Japan; 5 Department of Data Science, The Institute of Statistical Mathematics, Tachikawa, Tokyo, Japan; University of California, Los Angeles, United States of America

## Abstract

Functional fluorescence imaging has been widely applied to analyze spatio-temporal patterns of cellular dynamics in the brain and spinal cord. However, it is difficult to integrate spatial information obtained from imaging data in specific regions of interest across multiple samples, due to large variability in the size, shape and internal structure of samples. To solve this problem, we attempted to standardize transversely sectioned spinal cord images focusing on the laminar structure in the gray matter. We employed three standardization methods, the affine transformation (AT), the angle-dependent transformation (ADT) and the combination of these two methods (AT+ADT). The ADT is a novel non-linear transformation method developed in this study to adjust an individual image onto the template image in the polar coordinate system. We next compared the accuracy of these three standardization methods. We evaluated two indices, i.e., the spatial distribution of pixels that are not categorized to any layer and the error ratio by the leave-one-out cross validation method. In this study, we used neuron-specific marker (NeuN)-stained histological images of transversely sectioned cervical spinal cord slices (21 images obtained from 4 rats) to create the standard atlas and also to serve for benchmark tests. We found that the AT+ADT outperformed other two methods, though the accuracy of each method varied depending on the layer. This novel image standardization technique would be applicable to optical recording such as voltage-sensitive dye imaging, and will enable statistical evaluations of neural activation across multiple samples.

## Introduction

Various imaging techniques have been developed to evaluate spatial activity patterns of the brain in the last two decades. Among these techniques, functional magnetic resonance imaging and positron emission tomography have been widely applied to the mapping of neural activation in the human brain. Neural activation can be detected using regression analysis or cross-correlation analysis for the brain; however there had been a difficulty in integrating spatial information on neural activation across multiple samples due to inter-sample variability in the brain shape and size. In order to solve this problem, projection of individual imaging signals onto the standard brain atlas has been attempted, and several methods of anatomical standardization for the brain have been proposed, for example, point-based standardization (e.g., Human Brain Atlas [1], [2]) and intensity-based standardization (e.g., Statistical Parametric Mapping Method [3], [4], Michigan Method [5]). These standardization methods enable statistical tests for the group analysis across subjects and activation mapping in the standard brain atlas with statistical values. In addition, various non-linear transformation methods for registration have been proposed [6]–[12]. The purpose of such a method is to match different images of a particular subject. Some standardization methods have been suggested not merely for brain but also for the liver or breast [13]–[16]. In animal studies, fluorescent voltage-sensitive dye (VSD) imaging has been widely applied to analyze spatio-temporal patterns of neural electric activities in the brain and spinal cord [17]–[24]. However, neither a registration nor standardization method for VSD imaging has ever been proposed for the brain or spinal cord of animals. In the present study, we attempted to develop a method that could be applied to projection of VSD fluorescent signals in a transversely sectioned spinal cord onto the standardized atlas, matching laminar structures of the gray matter. The primary purpose of our standardization of spinal cord images is, firstly, to delineate the white and gray matters and secondly, to match internal structures to laminas I-X in the gray matter. However, it is difficult to distinguish each lamina in the gray matter on the background of the black and white image for VSD imaging in which boundaries of the internal anatomical structure is not visibly clear, although the position of the central canal can be barely identified in it. Further, the pixel intensity and spatial distribution pattern are varied across samples. Therefore, the intensity-based transformation method, which adjusts spatial distribution pattern of an individual image to that of the template image, is not appropriate to apply to the background picture of VSD imaging. The alternative approach is point-based linear transformation, which adjusts the position of characteristic points in individual images to those in the template image. However, sufficient accuracy of standardization by this method is hardly expected, because the outline of the spinal cord does not provide enough information to define characteristic points due to the simplicity of the outline curve. Therefore neither the intensity-based nor point-based standardization method is applicable to VSD imaging. Considering these limitations, we must develop a new standardized method depending on only some keystones defined by the outline of the spinal code and the position of the central canal. The ordinary method for image transformation is the affine transformation (AT), which projects individual images onto template images through linear combinations of translation, rotation, scaling, and shearing operation. The AT method corrects keystone distortions and adjusts scales of individual images to the template images. However the accuracy of the projection by the AT method is not sufficient, because the AT does not correct local distortion appropriately, although it does work for global distortion correction.

Here, we propose a nonlinear transformation method, which corrects scaling of the radial direction from the origin of the polar coordinate system set on the image because of the limitation of linear combination of translation. Hereafter, we call this method the angle dependent transformation (ADT). The ADT method is a contour-based standardization method. It adjusts the shape of the contour of individual image to the template image. By this method, the characteristics of the contour shape can be represented not as a set of points but as a continuous outline function, and thus we can expect higher performance of standardization.

In this study, we used neuron-specific nuclear protein (NeuN)-stained histological pictures of the transversely-sectioned spinal cord, which are capable of distinguishing the laminar structure of the spinal cord. We then evaluated the accuracy of the ADT, by comparing the performance of three standardization methods, the AT, ADT, and the combination of these two methods (AT+ADT). For the comparison we applied two indices, i.e., the ratio of the pixels which were correctly classified to each lamina and the error ratio using the leave-one-out cross validation method.

## Materials and Methods

### Data acquisition

**Figure 1:**
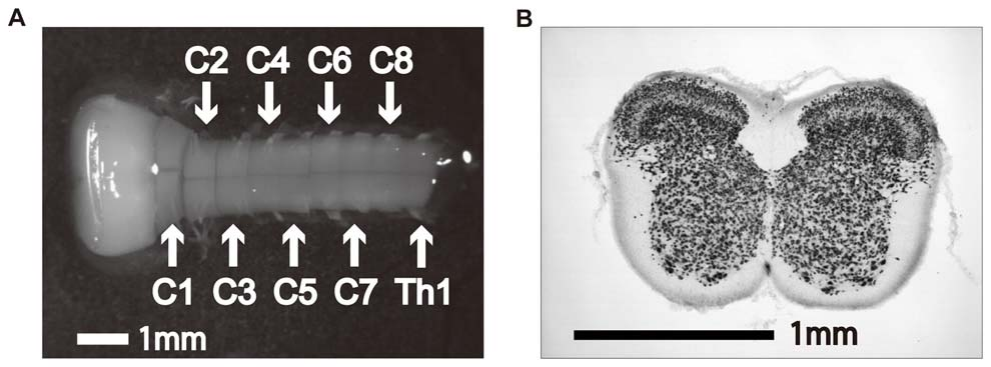
Sample images of spinal cord. (**A**) Isolated lower brainstem and cervical spinal cord. (**B**) Neuron-specific marker NeuN-stained histological image of the cross-section of the spinal cord at C4 level. A NeuN-stained image showed clearly demarcated laminar structures of the gray matter of the spinal cord.

We prepared benchmark images, which were obtained from NeuN-stained histological pictures of the transversely-sectioned cervical spinal cord. The procedures using animals were done in accordance with the NIH Guide for the Care and Use of Laboratory Animals and were approved by the Animal Research Committee of Shimane University School of Medicine. The histological technique of NeuN staining has been described elsewhere [17]. Briefly, neonatal rats (Wistar, 4–5 day-old, n = 4) were deeply anesthetized by intraperitoneal injection of chloral hydrate (350 mg/kg), perfused transcardially with saline, and fixed with 4% paraformaldehyde in 0.1 M phosphate buffer (pH 7.3). The spinal cords were removed and post-fixed with same fixative. Subsequently, several (mostly 5–8) sections per single spinal cord with 40 μm thickness were cut out mainly from the 4th cervical segment (C4) on a freezing microtome. The sections were incubated overnight in mouse anti-NeuN (Chemicon/Millipore) diluted 1∶500 by phosphate buffered saline (pH 7.3) containing 0.2% Triton X-100 and 3% normal donkey serum. Subsequently, the sections were incubated in biotin-conjugated anti-mouse IgG (1∶500; Jackson) for 3 h, incubated in avidin-biotin-peroxidase complex (1∶100, Elite Kit, Vector) for 1 h, and then developed in 25 ml of PB containing 10 mg diaminobenzidine and 10 μl of 30% H_2_O_2_. After NeuN was immunohistochemically stained, we excluded 3 damaged sections, and finally obtained 21 images. The format of each histological image was RGB, and the resolution and size were 72dpi and 1,280*960 pixels, respectively (Figure 1).

### Transformation methods of individual image onto template

The outline of the histological image of transversely-sectioned spinal cord and the boundary of each lamina in the gray matter were traced by a single experienced neuroanatomist for each sample. We confirmed that this manual tracing yielded highly reproducible results. The outline curve was smoothed with the 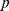-th order Fourier series in order to reduce the jags caused by the trembling of hand during tracing process.

**Figure 2:**
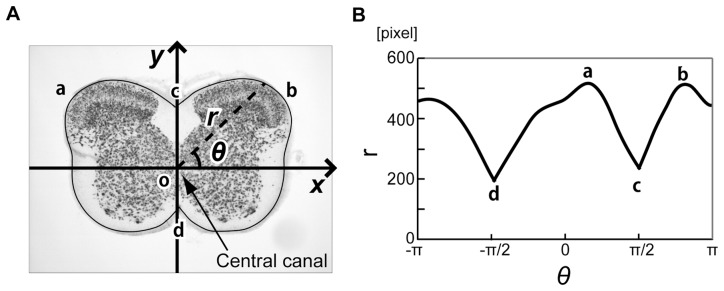
Extraction of the outline based on the polar coordinate system. (**A**) A schematic drawing of the extraction of the outline. (**B**) A development of the smoothed outline in 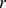-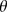 space. **a** and **b** indicate the maximum points, and **c** and **d** indicate the minimum on the outline function in the 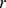-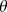 space, and these points were used as ground control points (GCPs) to estimate the parameters for the AT.

We selected the position of the central canal, which is a conspicuous anatomical landmark located in the center region of the spinal cord, as the origin of the axis in the polar coordinate system. The radius toward the outline and the angle from x-axis were denoted by 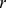 and 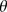, respectively (Figure 2A). Every radius meets the outline at a point; therefore the outline curve can be represented with a univariate function.

The 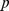-th order Fourier series is defined as
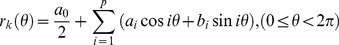
(1)


**Figure 3:**
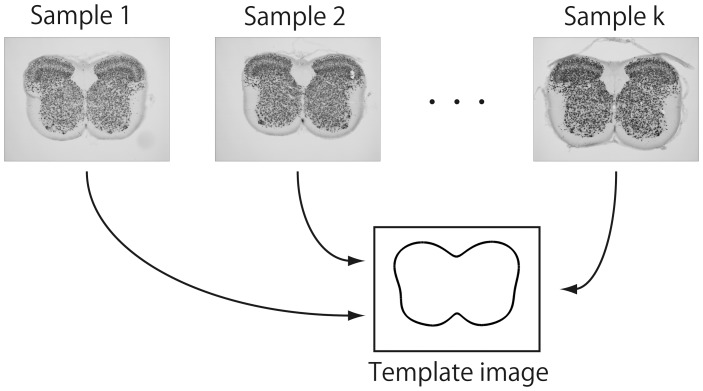
A schematic drawing of the standardization process. Individual sample image was projected onto the template image through the affine transformation (AT), angle-dependent transformation (ADT) and combination of these two methods (AT+ADT).

Here, 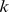 is an index of sample. Figures 2A and 2B show a representative smoothed outline function and its development in the 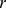-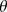 space, respectively. The smoothed outline function of each sample could be projected onto the outline of template image (Figure 3) through the AT, ADT and combination of these two methods (AT+ADT). Although the template images of an animal brain, such as baboon [25], macaque [26] and rat [27], have been developed, no template image for the rat spinal cord has yet been proposed. Then we prepared a template image of the rat spinal cord. The procedure for obtaining template image is explained in Appendix S1.

### Affine transformation (AT)

An affine transformation is a combination of linear transformations that preserves collinearity and ratios of distances. A matrix representation of affine transformation of the *n*-th input pixel 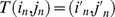 is given by
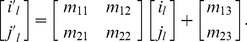
(2)


The parameters can be optimized by the least square method. An affine transformation requires at least three predefined ground control points (GCPs) to determine the six parameters by least square methods. It is desirable to keep a sufficient distance between GCPs to maintain the optimization process stable.

Suppose 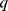 are GCPs selected on the outline of the image of each sample and template image, then the least square error will be
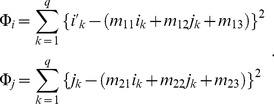
(3)


Taking the derivative of 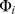
with respect to these parameters and setting them to zero gives the following set of normal equations:
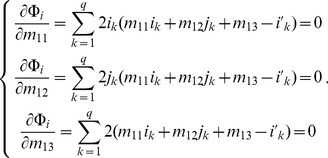
(4)


Solving these equations, the parameters 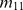, 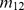 and 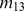 can be optimized, and applying this procedure for 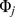 gives the optimized parameters 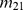, 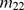 and 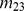. Using these estimated parameters, any point on an image of individual sample can be projected onto the template image. Forward transformation defined as (2) may cause two problems as a computational procedure depending on the specific spatial transform function. Some output pixels may not be covered and many source pixels can be mapped to the same output pixel. In order to overcome these problems with the forward mapping, many image spatial transform implementations use inverse transformation 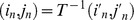, which determines the corresponding location in the input image. An approximate value for the input image at 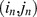 can be obtained by interpolation and will be the value for the n-th pixel in the transformed image. We used the bi-linear interpolation method using the pixels nearest to 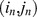.

### Angle-dependent transformation (ADT)

The angle-dependent scaling parameter 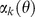 can be precisely obtained with the ratio 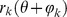 to 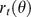 for each value of 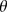


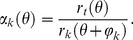
(5)


Then 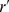
and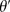 in the new image are denoted by:
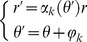
(6)


The coordinate transformation from the Cartesian coordinate system to the polar coordination system for the points 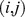
and 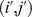 are realized as



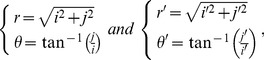
(7)




respectively.

Substituting (7) into (6) gives the relation between 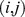
and 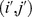


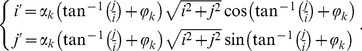
(8)


The coordinate values 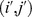 were corrected using the bi-linear interpolation method as same as for the AT.

In this study, we employed the AT for the correction of keystone distortion, which is caused when image acquisition with a camera was performed obliquely to the sample surface. We assumed that the inter-sample variation of the shape was due to a non-linear local distortion and attempted to correct it using the ADT as much as possible. Therefore we sequentially applied the ADT after the AT for the AT+ADT method. All algorithms for standardization were implemented in Matlab R2008b (MathWorks, MA, USA) and ran on a Pentium-based Windows 7 computer.

## Results

The outline curve was smoothed by Fourier series (1). It is important to choose a sufficiently large value for the order of Fourier series in the eq.(1), lest any relevant correlations in the data be missed. On the other hand, too large orders may cause over-fitting problems and deteriorate the smoothness.

We repeated the numerical analyses with 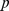 values incremented from 1 to 40 and found that for 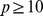 the results remained essentially unchanged and the outline function was sufficiently smoothed.

There have been a number of proposed methods for model selection. Among the methods, Akaike Information Criteria (AIC) is the one which has been popularly used [28]. The order of Fourier series 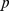 can be optimized using AIC. In this study we attempted the analysis with changing the value of p, and found that similar results were yielded with 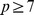. Therefore we conclude that for our data an order of 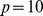 represents a good compromise between the extremes.

For the AT, the number and location of GCPs have to be decided. We selected two local maximum points in the region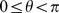, two local minimum points on the outline of the image in the 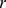-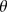 space, and the origin of the polar coordinate system (totally five GCPs) (Figure 2). In the case we further included two more local maximum points in the region 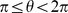 for GCPs, it deteriorated the performance of lamina discrimination. Therefore we selected these GCPs for the analyses.

**Figure 4:**
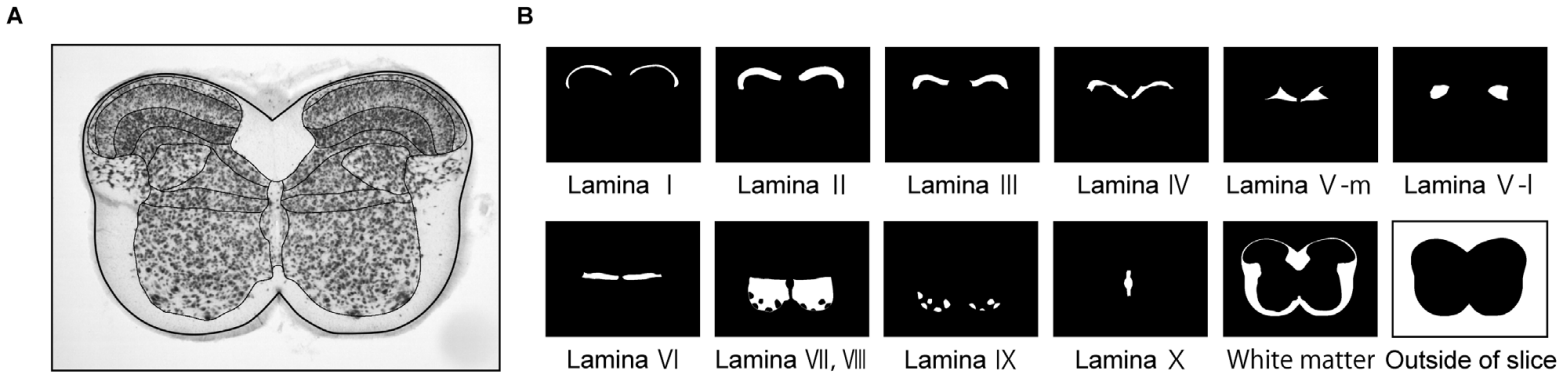
Conversion of a histological image to binary images of laminar structure. (**A**) Histological image data of the cross-section at the 4th cervical spinal cord. The boundaries of laminas in the gray matter were demarcated by solid lines. (**B**) Binary images corresponding to each lamina and the white matter. The pixels which belong to a lamina were set to 1 and blotted out in white, and the other pixels were blotted out in black.



To investigate the layer discrimination ability of each method, we prepared binary image for each lamina and sample (Figure 4). The value of a pixel 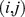 in the binary image is defined as

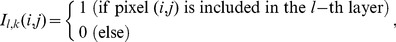
(9)here, l and k are indices of lamina and sample, respectively. The value of a pixel in the transformed image is similarly defined as 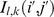.

We averaged binary images across samples for each layer as follows:
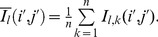
(10)


**Figure 5:**
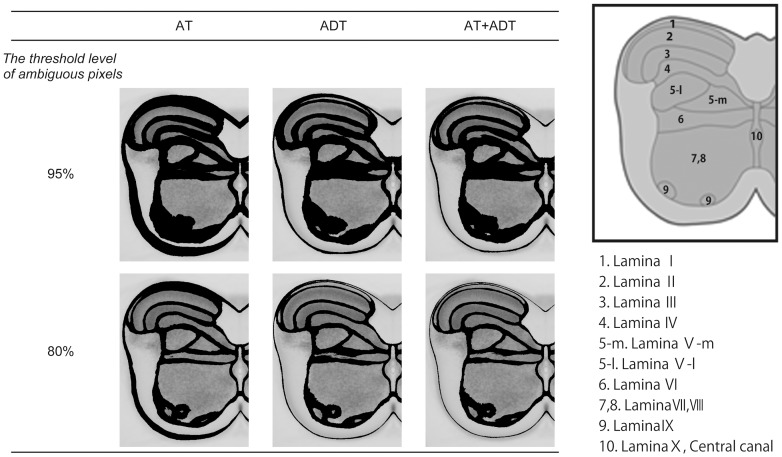
Spatial distribution of categorized pixels and ambiguous pixels. Black colored regions represent ambiguous pixels that were categorized with the probability less than 95% (upper) and 80% (bottom) threshold level, respectively. In this figure, we can overview the spatial precision of each transformation method.

The mean value for a pixel must vary between 0 and 1 and indicates the probability that a pixel in the input image belongs to 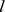-th layer in the template image. Hereafter the percentage of the mean value will be called the frequency ratio. Suppose the frequency ratio achieves 100, a pixel 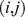 in the input image would be transformed exactly into the same layer in all samples. On the contrary, suppose the frequency ratio is 0, a pixel 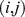 in the input image would be transformed into a different layer, the white matter, or outside the slice in all samples. Therefore the frequency ratio is an appropriate index to evaluate the performance of these transformation methods, and the spatial distribution of frequency ratios constitutes a frequency ratio map. The pixels in the frequency ratio map whose ratios were lower than the threshold level were regarded as ambiguous pixels, that is, these pixels did not belong to any layers. We illustrated the spatial distribution map of ambiguous pixels with a threshold level at 95%. Subsequently the threshold was decremented from 95% to 50% by 5%, and we found that the area size, which did not belong to any layers, increased until the threshold level decremented to 80% and keeps nearly the same value with threshold levels lower than 80%. Figure 5 illustrates the spatial distribution of categorized pixels and ambiguous pixels with threshold levels 95 and 80%.

Table 1 shows the number of pixels in each layer in the transformed image, which belonged to the corresponding layers in the input image. The total sum of categorized pixels across all layers was the least in the AT method for both threshold levels 95% and 80%. In other words, the AT method had the poorest accuracy among the methods in terms of categorization. Large differences in the accuracy of categorization were recognized in laminas I, II, III, IV, VI and IX. The pixels in laminas I and IX were poorly categorized by the AT method regardless of thresholds selected. In contrast, the number of categorized pixels in lamina VI was least in the ADT method. While there were few differences in the region close to the central canal, there were large differences in regions that are far from the central canal (see Figure 5).

**Table 1:** Number and percentage of pixels belonging to each lamina in the transformed image.

Threshold levelof ambiguous pixels	95%						80%					
Transformation method	AT		ADT		AT+ADT		AT		ADT		AT+ADT	
**Lamina I**	**32**	**0.01%**	3570	0.68%	5055	0.97%	**1379**	**0.26%**	8886	1.70%	9878	1.89%
**Lamina II**	**25973**	**4.98%**	34227	6.57%	33510	6.43%	**36397**	**6.98%**	42756	8.20%	41540	7.97%
**Lamina III**	**10504**	**2.01%**	13945	2.67%	14722	2.82%	**19606**	**3.76%**	22835	4.38%	22197	4.26%
**Lamina IV**	**8707**	**1.67%**	12299	2.36%	12077	2.32%	**20063**	**3.85%**	23694	4.54%	22570	4.33%
**Lamina V-m**	5471	1.05%	**4852**	**0.93%**	5422	1.04%	**8250**	**1.58%**	9189	1.76%	8888	1.70%
**Lamina V-l**	11517	2.21%	12248	2.35%	**11024**	**2.11%**	**16303**	**3.13%**	17231	3.31%	16859	3.23%
**Lamina VI**	3163	0.61%	**2702**	**0.52%**	3121	0.60%	13852	2.66%	**10535**	**2.02%**	13715	2.63%
**Lamina VII, VIII**	96135	18.44%	99707	19.12%	**94886**	**18.20%**	113158	21.70%	119280	22.88%	**112556**	**21.59%**
**Lamina IX**	**0**	**0.00%**	503	0.10%	377	0.07%	**1696**	**0.33%**	2784	0.53%	2350	0.45%
**Lamina IX**	**3947**	**0.76%**	4842	0.93%	4751	0.91%	**5467**	**1.05%**	6309	1.21%	5977	1.15%
**White matter**	**89097**	**17.09%**	121620	23.33%	120398	23.09%	**118455**	**22.72%**	142201	27.28%	138002	26.47%
**Total of categorized pixels**	**254546**	**48.82%**	310515	59.56%	305343	58.57%	**354626**	**68.02%**	405700	77.82%	394532	75.67%
**Inside of the outline template**	521351	100.00%	521351	100.00%	521351	100.00%	521351	100.00%	521351	100.00%	521351	100.00%

The bold type indicate the method which gave the smallest number of pixels for each layer.

Medial and lateral portions of lamina V were classified into V-m and V-l, respectively.

Additionally we statistically evaluated the categorization ability of each method by the leave-one-out cross validation. We left out one of histological images among 42 images and labeled it as k-th image, and produced a frequency map with remained 41 images by the same procedure that was explained above. Then, to compare with the left out image, the frequency ratio map was converted into a binary image of each lamina or white matter. For preparing the binary image, the values of the pixels which belonged to the layer with highest frequency ratio were replaced by 1, and others were set to be 0. Then, the produced binary image was inversely transformed by the AT, ADT and AT+ADT methods, and the parameters were optimized so that it fitted the left out image as much as possible. Then we computed the error ratio to evaluate how much the inversely transformed image agrees with the left out image.

**Figure 6:**
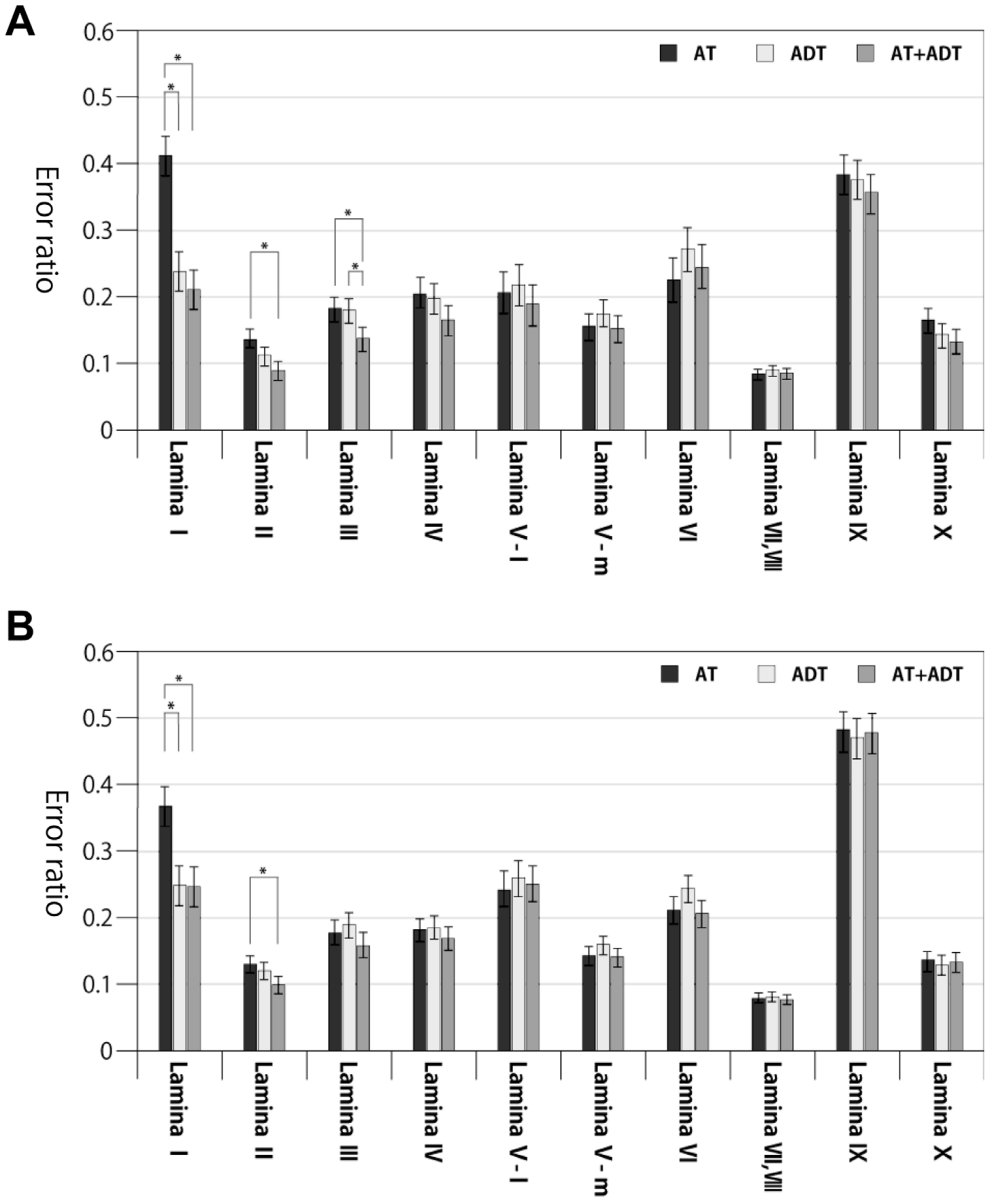
Comparisons of the mean error ratio estimates for type 1 error (A) and type 2 error (B). The significance of the differences were evaluated by two-way ANOVA with Bonferroni's post hoc test, and results with significance level 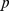
<0.05 (Bonferroni-corrected 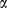
<0.05/3 = 0.017) were marked with “*”.

Pixels, which were categorized in each lamina in the transformed image but were not categorized in the left out image, induce type 1 error. In the opposite case, pixels induce type 2 error. Then, type 1 and 2 error ratios can be defined as 
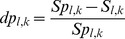
 and 
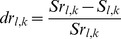
 respectively, where 
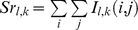
 and 
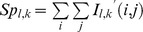
 (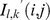: the pixel value at a coordinate 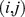
in the 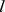-th lamina of the 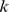-th image) are total numbers of pixels which satisfy conditions 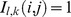 and 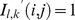, respectively. Repeating this procedure for all images (k = 1,..n) yields mean error ratio estimates 
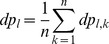
 and 
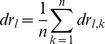
 for type 1 and 2 error, respectively. Two-way analysis of variance (ANOVA) was performed to assess mean error ratio differences due to the methods (AT, ADT and AT+ADT) and laminas for type 1 and 2 error. There were significant main effect of the methods (type 1: F(2,1230) = 26.77, 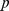
<0.01, type 2: F(2,1230) = 13.14,
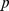
<0.01) and laminas (type 1: F(9,1230) = 77.96, 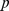
<0.01, type 2: F(9,1230) = 119.39, 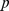
<0.01) and also interaction (type 1: F(18,1230) = 7.72, 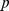
<0.01, type 2: F(18,1230) = 5.33, 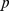
<0.01). It indicates that the performance of the methods is different depending on the laminas. Subsequently the difference of mean error ratios corresponding to the method for each lamina, which satisfy the significance level, were evaluated by the paired 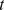-test with the Bonferroni correction (Figure 6).

In the case of type 1 error ratio, the error ratio of the AT+ADT method was significantly lower than that for the AT method in lamina I, II and III. A tendency that the AT+ADT gave the lowest error ratio in laminas IV, V-l, IX and X was seen, although it did not satisfy the significance level. In the case of type 2 error ratio, the AT+ADT method showed a lower error ratio than the AT method in laminas I and II. There was no significant difference among the methods in other laminas. The AT+ADT method never gave the highest error ratio in any lamina for both type 1 and 2 error ratios.

## Discussion

In this study, we compared the performance of three image standardization methods, the AT, ADT and AT+ADT in terms of laminar categorization, and found that the performance varied depending on the location of lamina. The ADT method showed higher performance than the AT method in the accuracy of transformation from each individual image to the template image and sufficiently reduced type 1 and 2 error ratios in lamina I. Similar tendency was recognized in lamina II. Since the ADT method transforms pixels by adjusting the angular-dependent scaling, higher performance is expected in the laminas, which lie along with the circumferential direction. However, the keystone distortion is not corrected by the ADT method alone. Therefore, the AT method should be applied before applying the ADT method. The method AT+ADT improved the performance in laminas I, II, III and IV; this was most likely because the combined method canceled disadvantages of each method.

We defined the outline of the spinal cord sample not by automated procedure using an image processing technique but by manual procedure, because it was difficult to extract the outline of the spinal cord on VSD images due to low spatial resolution of the VSD images. The development of an algorithm to automate the procedure will be for a further study.

**Figure 7:**
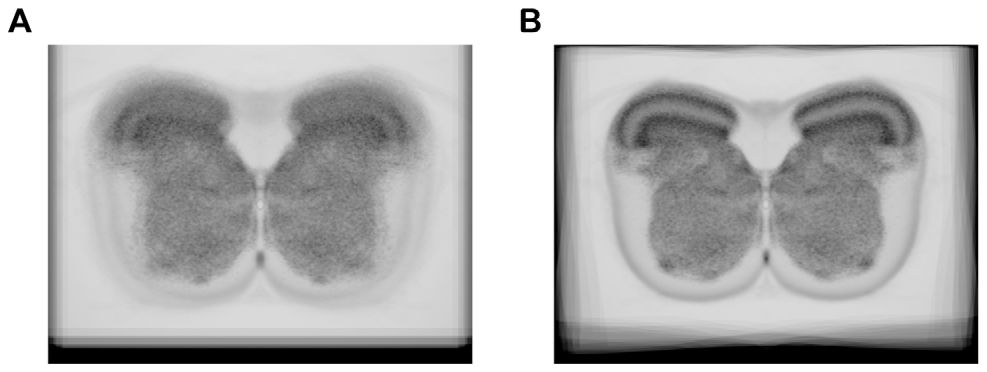
Spatial averaging of histological images. (**A**) Averaged histological image without standardization. It was constructed by averaging all histological images to adapt to the position of the central canal. (**B**) Standardized anatomical image. All histological images were transformed using the AT+ADT method, and the averaged image was constructed from them.

Since the central canal was selected as the origin of the polar coordinate for the ADT method and a GCP for the AT method, pixels close to the central canal would not be transformed into distant locations. This would be a reason why there was no significant difference among methods in the medial portion of lamina V and in laminas VII, VIII and X. The type 1 and 2 error ratios in laminas VII and VIII were lower than those in other laminas, because the size of laminas VII and VIII is much larger than that of other laminas. In the case of lamina IX, the type 1 and 2 error ratios were much higher than other laminas in all transformation methods. This was attributed to its area size and location that are smaller and more variable as compared with those of other laminas. Nevertheless, the area of lamina IX could be successfully categorized, although the boundary of the area was ambiguous in individual input images (Figure 5). By averaging standardized images, the internal structure appeared remarkably clear (Figure 7).

Fluorescent VSD imaging has become a popular technique to record global electric activities in the brain and spinal cord. VSD imaging techniques have been applied, e.g., to the sensory system of the spinal cord [17]–[19] as well as to the respiratory central pattern generator of the brainstem [20]–[23]. Also, mathematical methods to more efficiently detect neural activities from spatio-temporal data obtained by VSD imaging have been developed [23], [24]. However, it has been difficult to associate the region of neural activation detected by VSD imaging with the anatomically defined site, e.g., with a distinct lamina in the spinal cord. This study will provide a solution for this problem. When applying this method to the brain, it is possible to define the brain structure located in the midline as the central point instead of the central canal. Specifically, the midline of the fourth ventricle floor in the medulla oblongata and pons, the aqueduct of Sylvius in the midbrain and the midline of the third ventricle floor in the diencephalon could be defined as the central point, respectively.

In summary, standardization methods by which individual images are projected onto a common platform image, i.e., the standard spinal cord atlas, were presented, and the AT+ADT combined method most reliably associates the region of neural activation with the location of the lamina. This novel image standardization technique would be applicable to optical recording such as VSD imaging, and will enable statistical evaluations of neural activation across multiple samples.

## Supporting Information

Appendix S1 **Template image preparation.**
(DOC)

Figure S1 **Outline functions.** (**A**) Raw functions, (**B**) rigid-body transformed functions and (**C**) the template function.
(TIF)
