# Supplementary material for: Standardization of Size, Shape and Internal Structure of Spinal Cord Images: Comparison of Three Transformation Methods
Source: PLoS One. 2013 Nov 5;8(11):e76415. doi: 10.1371/journal.pone.0076415 (PMC3818318; doi:10.1371/journal.pone.0076415)
Supplement: Appendix S1 — Template image preparation. (DOC) [file pone.0076415.s001.doc]

**Appendix S1**

**Template image preparation**

We used histological image of transversely sectioned spinal cord obtained from 21 samples and also its mirror images to form the template image symmetric (totally n=42 images). The outline of the spinal cord was smoothed by 10-th order Fourier series with period $2\pi$ for each sample. One image out of 42 images was preserved for a target image. The smoothed outline function for each sample was projected onto the target image through the transformation method as explained below. The rotation in the polar coordination corresponds to the translation along $\theta$ axis in the $r$-$\theta$ space. Let the smoothed outline function of the target image, $k$-th image and rotation parameter be denoted $r'\text{(}\theta\text{)}$, and $\phi_{k}$ respectively. The rotation parameter $\phi_{k}$ can be optimized by minimizing the error variance between $r'\text{(}\theta\text{)}$ and (Figures 8A,B). The scale parameter for $k$-th image $\alpha_{k}$ was defined as a ratio of mean length of the radius of $r_{k}\text{(}\theta\text{)}$ to $r'\text{(}\theta\text{)}$;

$\alpha_{k}=\frac{\int_{0}^{2\pi} r'\text{(}\theta\text{)}d\theta}{\int_{0}^{2\pi} r_{k}\text{(}\theta\text{)}d\theta}$. (14)

Then, averaging the rigid-body transformed outline function across sample yields an outline function of the template image as

. (15)

Although the scale of the constructed template image depends on the selection of the target image, it does not change its shape. Figure S1 shows the outline function of the template image. The inner structure of transversely sectioned spinal cord for $k$-th image can be projected onto the target image with the same optimized rotation and scaling parameter. Then a target image with inner structure can be constructed by averaging the projected images across samples.
