# Supplementary figures and images for: Standardization of Size, Shape and Internal Structure of Spinal Cord Images: Comparison of Three Transformation Methods
Source: PLoS One. 2013 Nov 5;8(11):e76415. doi: 10.1371/journal.pone.0076415 (PMC3818318; doi:10.1371/journal.pone.0076415)

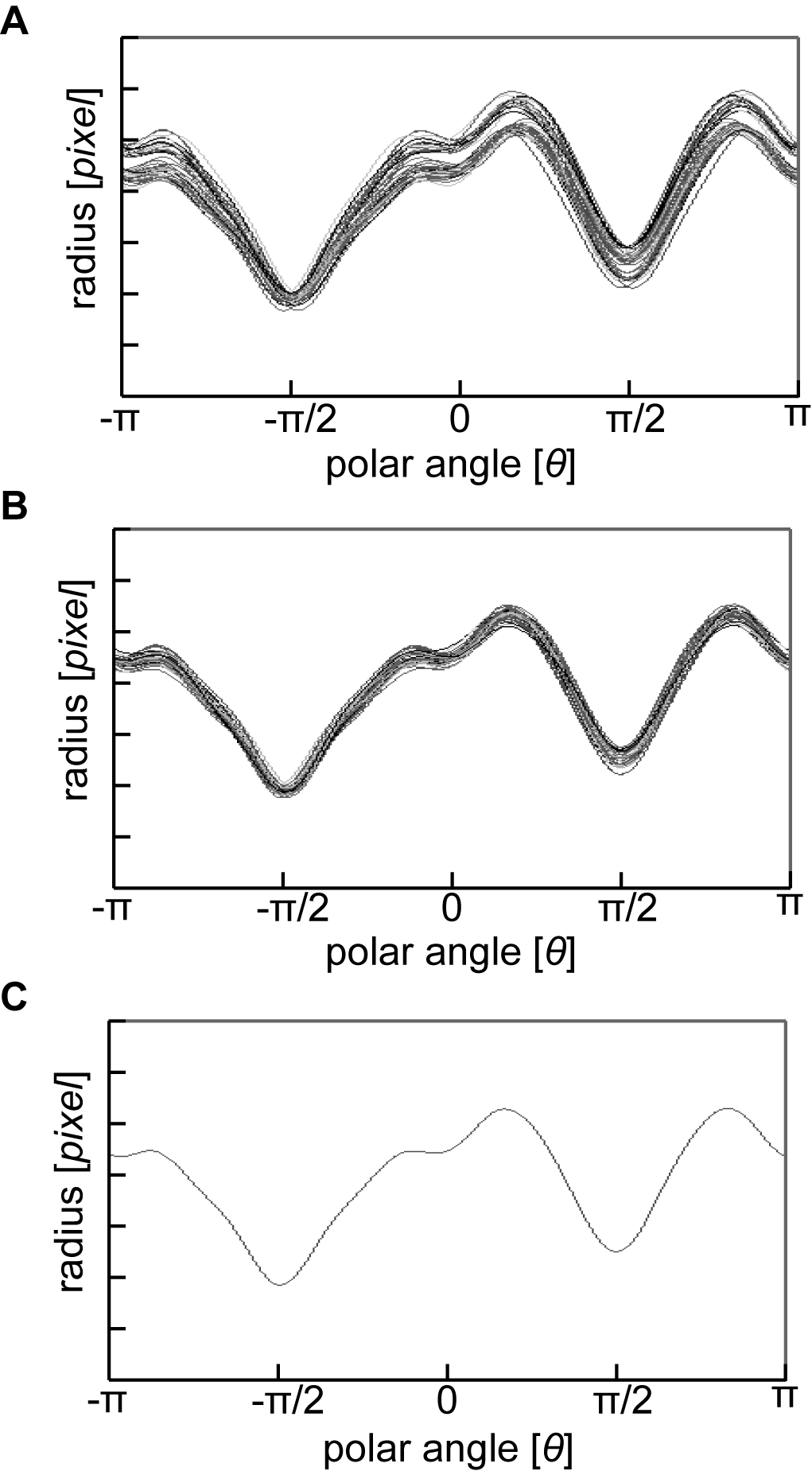

Supplement: Figure S1 — Outline functions. (A) Raw functions, (B) rigid-body transformed functions and (C) the template function. (TIF) [file pone.0076415.s002.tif]
